# Altered β1,6-GlcNAc branched *N*-glycans impair TGF-β-mediated Epithelial-to-Mesenchymal Transition through Smad signalling pathway in human lung cancer

**DOI:** 10.1111/jcmm.12331

**Published:** 2014-06-09

**Authors:** Na Li, Haineng Xu, Kun Fan, Xijun Liu, Jingjing Qi, Chao Zhao, Peng Yin, Liying Wang, Zengxia Li, Xiliang Zha

**Affiliations:** a Department of Biochemistry and Molecular Biology, Shanghai Medical College, Fudan UniversityShanghai, China; b Key Laboratory of Glycoconjugate Research, Ministry of HealthShanghai, China; c Key Laboratory of Molecular Medicine, Ministry of EducationShanghai, China; d Key Laboratory of Stem Cell Biology, Institute of Health Sciences, Shanghai Institutes for Biological Sciences, Chinese Academy of Sciences/Shanghai Jiao Tong University School of MedicineShanghai, China; e Key Laboratory of Cell Biology, Institute of Biochemistry and Cell Biology, Shanghai Institutes for Biological Sciences, Chinese Academy of SciencesShanghai, China

**Keywords:** GnT-V, *N*-glycans, lung cancer, EMT, TGF-β1

## Abstract

The change of oligosaccharide structure has been revealed to be crucial for glycoproteins' biological functions and cell biological characteristics. *N*-acetylglucosaminy transferase V (GnT-V), a key enzyme catalysing the reaction of adding β1, 6-*N*-acetylglucosamine (GlcNAc) on asparagine-linked oligosaccharides of cell proteins, has been implicated to a metastastic-promoting oncoprotein in some carcinomas. However, this correlation might not be subjected to all types of cancers, for example, in non-small cell lung cancers, low level of GnT-V expression is associated with relatively short survival time and poor prognosis. To explain the role of GnT-V in lung cancer progression, we studied the association of GnT-V expression with lung cancer EMT behaviour. We found that GnT-V expression was correlated with epithelial marker positively and mesenchymal marker negatively. GnT-V levels, as well as β1,6-GlcNAc branched *N*-glycans, were strongly reduced in TGF-β1-induced EMT of human lung adenocarcinoma A549 cells. Further studies showed that suppression of β1,6-GlcNAc branched *N*-glycans by inhibitor or GnT-V silencing in A549 cells could promote TGF-β1-induced EMT-like changes, cell migration and invasion. Meanwhile, overexpression of GnT-V impaired TGF-β1-induced EMT, migration and invasion. It suggests that GnT-V suppresses the EMT process of lung cancer cells through inhibiting the TGF-β/Smad signalling and its downstream transcription factors in a GnT-V catalytic activity–dependent manner. Taken together, the present study reveals a novel mechanism of GnT-V as a suppressor of both EMT and invasion in human lung cancer cells, which may be useful for fully understanding *N*-glycan's biological roles in lung cancer progression.

## Introduction

The change of oligosaccharide structure has been revealed to be crucial for biological functions of glycoproteins. These oligosaccharides on glycoproteins often play important roles in the regulation of the biological characteristics of tumours, especially in regulation of tumour invasion and metastasis 1. Each oligosaccharide of glycoproteins is synthesized *via* catalysis by a special glycosyltransferase, the expression of which affects specific functions of glycoproteins through glycosylation. Most of the cancer-associated changes of oligosaccharide structure of glycoproteins are because of the abnormal expression of glycosyltransferase genes, such as *N*-acetylglucosaminyltransferase V (GnT-V, also known as Mgat5) 2–4.

GnT-V catalyses the linkage of a GlcNAc to a core mannose of *N*-glycan to produce the GlcNAc-β1,6-Man branch to form tri-or tetra-antennary *N*-linked oligosaccharide chains 5. In the Golgi compartment, GnT-V transfers a GlcNAc residue onto growing *N*-glycans of integrins, cadherins, growth factor receptors (GFRs) and cytokine receptors, so that subsequent glycosylation results in formation of ‘multi-antennary’ chains 6.

Some studies demonstrate that GnT-V expression and its products β1,6-GlcNAc branched *N*-glycans are commonly increased in malignancies, for example, in human mammary, colon and glial tumours. This increment of *N*-glycan branching is considered to be positively associated with tumours malignant transformation 2,7–10. However, these correlations do not apply to all types of tumours. In the case of hepatocellular carcinoma, it was reported that low level or absence of GnT-V expression is related to high recurrence rate 10. Moreover, GnT-V and its product of β1,6-GlcNAc branched *N*-glycans are closely related to low malignant potential and good prognosis of the patients in bladder, neuroblastoma, gastric and lung cancers 11–14. These reports suggest that the clinical implication of GnT-V expression may differ in each kind of cancer, and the role of GnT-V in cancer progression could be tissue-specific. The functions of GnT-V and its products in human lung cancer progression, especially metastatic dissemination, remain to be investigated.

The epithelial-to-mesenchymal transition (EMT), which is characterized by the loss of epithelial adhesion and gain of mesenchymal features, is a fundamental biological process of embryonic development and cancer invasion and metastasis 15,16. The invasive nature of each tumour subtype is dependent on epithelial cells increasing their capacity for migration through EMT. EMT can be induced or regulated by various growth and differentiation factors. Transforming growth factor-β (TGF-β), besides playing the important roles in the control of cell proliferation, differentiation, apoptosis and ageing, is one of the established EMT inducer 17–19.

Transforming growth factor-β functions by binding to type II TGF-β receptors (TβRII), transphosphorylation of type I TGF-β receptors (TβRI), and subsequent phosphorylation of Smad2 and Smad3. Phosphorylated Smad2/3 forms a trimer with Smad4 that then translocates to the nucleus and interacts with transcription factors, co-activators and co-repressors to suppress epithelial genes and promote the expression of mesenchymal proteins 20. In addition, non-Smad signalling through activation of the mitogen-activated protein (MAP) kinases (e.g. p38, ERK1/2 and JNK), the small GTP-binding proteins (e.g. Ras, Rho and Rac1) and the cell survival mediators (e.g. PI3K, AKT and mTOR) has also been implicated in TGF-β-induced EMT 21. Furthermore, TGF-β signalling has extensive crosstalk with integrins/FAK signalling pathway 22.

There is evidence suggesting that the process of TGF-β-induced EMT can be regulated by various glycosyltransferases 23. It is still unclear whether GnT-V and *N*-Glycan branching regulate the lung cancer progression and EMT behaviour. In this study, we demonstrated the relationship between the GnT-V and EMT behaviour in human lung cancer cells. The results suggest that GnT-V is a suppressive regulator of TGF-β in controlling EMT and cell migration mainly through enhancement of TGF-β/Smad signalling. These results reveal a distinct inhibitory role of β1,6-GlcNAc branched *N*-glycans in lung cancer invasive/metastatic potential.

## Materials and methods

### Cell lines and cell culture

The human non-small cell lung cancer (NSCLC) cell line A549 was purchased from the Cell Bank of Type Culture Collection of Chinese Academy of Sciences (Shanghai, China). The other NSCLC cell lines H1299, H1975, H460 and Human embryonic kidney 293T were kindly provided by Prof. Xinyuan Liu (Chinese Academy of Sciences, Shanghai, China). The cells were cultured in RPMI-1640 medium or DMEM supplemented with 10% foetal bovine serum (FBS) and 1% L-glutamine. All cells were cultured at 37°C in 5% CO_2_ humid atmosphere.

### Reagents and antibodies

Cell culture and transfection reagents were bought from Life Technologies (Carlsbad, CA, USA). Human recombinant TGF-β1 was from R&D Systems (Minneapolis, MN, USA). Monoclonal antibodies against E-cadherin and *N*-cadherin, Matrigel and Transwell of 0.8 μm were purchased from BD Biosciences (San Jose, CA, USA). Monoclonal antibodies against total-Smad2/3, phospho-Smad2 and Smad3, Vimentin, Snail and Slug were purchased from Cell Signaling (Beverly, MA, USA). Biotinylated L-PHA, E-PHA, WGA, GNA, ConA, DSA and Fluorescein Avidin D, R.T.U. Horseradish Peroxidase Streptavidin were products of Vector Laboratories (Burlingame, CA, USA). Dky X Rb IgG (H+L) Cy3, Gt Ms IgG FLUOR and swainsonine were purchased from Merck Millipore (Darmstadt, Germany). Rhodamine Phalloidin was from Cytoskeleton (Denver, CO, USA). Anti-GnT-V antibody was purchased from Abcam (Cambridge, MA, USA). Anti-GAPDH antibody, Anti-Histone1 and secondary antibodies conjugated with HRP were ordered from Kang-Chen Biotech (Shanghai, China).

### Kaplan–Meier Plotter online survival analysis

Kaplan–Meier Plotter online survival analysis was performed in a large combined lung cancer data set (www.kmplot.com/lung), which contains survival information of 1715 lung cancer patients and gene expression data obtained by using three different versions of Affymetrix gene microarrays 24,25. We used the online-available meta-analysis tool, to test possible correlations between expression of GnT-V (Affymetrix ID: 212098-at) with overall survival. Lung cancer patients were grouped by all stage (*n* = 1406) and stage I (*n* = 440), and tumour samples were equally grouped into low and high GnT-V expression based on the mRNA levels. Significant differences in overall survival time were assessed with the Cox proportional hazard (log-rank) test.

### Oncomine analysis

The Oncomine cancer microarray database (Compendia Bioscience, Ann Arbor, MI, USA), a repository for published cDNA microarray data (www.oncomine.org), was used to analyse the GnT-V and EMT markers' mRNA expression profiles of human lung cancer tissues relative to their normal controls. In this study, Bhattacharjee publicly available data sets of the gene expression profiles were chosen 26. mRNA expression profiles for GnT-V, E-cadherin, *N*-cadherin and Slug were evaluated by using Mgat5/31313-at, CDH1/2082-s-at, CDH2/2053-at and Snail2/38288-at respectively. mRNA expression levels were displayed by using log2 median-centred ratio boxplots for lung cancer *versus* normal. Standardized normalization techniques and statistical calculations are provided on the Oncomine website and published 27.

### RNA isolation and real-time PCR

RNA isolation, reverse transcription and real-time PCR (qRT-PCR) analysis were performed as previously described 28. Primers used in the qRT-PCR analysis were as follows: human GnT-V (NM-002410.3) forward 5′-GCACCGGAACAAACTCAACC-3′ and reverse 5′-CCATAG TCTGCGTAGCAGGG-3′; human E-cadherin (NM-004360.3) forward 5′-G CCCCGCCTTATGATTCTCTGC-3′ and reverse 5′-CTCGCCGCCTCCGTACATGTC-3′; human *N*-cadherin (NM-001792.3) forward 5′-CCACAG CTCCACCATATGACT-3′ and reverse 5′-CCCCAGTCGTTCAGGTAATC-3′; human Vimentin (NM-003380.2) forward 5′-CTCTTCCAAACTTTTCCT CCC-3′ and reverse 5′-AGTTTCGTTGATAACCTGTCC-3′; human Snail (NM-005985.3) forward 5′-TTCTTCTGCGCTACTGCTGCG-3′ and reverse 5′-GGGCAGGTATGGAGAGGAAGA-3′; human Slug (NM-003068.3) forward 5′-CGCCTCCAAAAAGCCAAAC-3′ and reverse 5′-CGGTAGTCCACAC AGTGATG-3′. A ratio relative to the GAPDH mRNA was used as a control in real-time PCR.

### Western blot and lectin blot

Cells were lysed in ice-cold lysis buffer containing SDS, protein assay buffer and blots were carried out as described previously 29. When protein-blotted polyvinylidene (PVDF) membranes (Merck Millipore) were prepared, and blocked with 5% BSA in TBS, the membrane was incubated with primary antibodies or biotinylated lectins in TBS buffer containing 0.1% Tween-20 (TBST) overnight at 4°C. The membrane was washed with TBS and probed with HRP-conjugated anti-rabbit or antimouse IgG (1:3000) or R.T.U. Horseradish Peroxidase Streptavidin for 1 hr at room temperature. After the membrane was washed with TBST, immunoreactive bands were visualized by using ECL reagents of Pierce (Thermo Fisher Scientific, Rockford, IL, USA).

### Flow cytometry analysis for cell surface *N*-glycans

Cultured cells were detached with 0.25% trypsin, and then collected by centrifugation at 300 g for 5 min. The cells were blocked with 1 mg/ml BSA in PBS, and successively incubated with biotinylated lectin-conjugated *N*-glycans of glycoprotein onto the cell plasma membrane for 1 hr at 4°C. All biotinylated lectin diluted to 1:250 with 0.5% BSA in PBS. Then the cells were incubated with Avidin D conjugated with Fluorescein at a dilution of 1:250 at 4°C for 45 min. in the dark. After washing with PBS, the cells were suspended in 300 μl PBS and analysed on the flow cytometry analysis (Beckman Coulter, Brea, CA, USA). A suspension of 1 × 10^4^ cells was analysed for each sample, and each experiment was repeated at least three times.

### Plasmids' construction, virus production and stable infection

Lentiviral recombinant vectors pCDH-puro-wtGnT-V and pCDH-puro-△cGnT-V were generated by PCR cloning the full length of human GnT-V (wtGnT-V) or C-terminal deletion mutant of GnT-V (△cGnT-V) from pcDNA3.1-GnT-V into pCDH-Puro, and were fully sequenced. The target sequence for GnT-V RNAi is 5′-CCTGGAAGCTATCGCAAAT-3′ (1508), and the scramble (control) sequence is 5′-GAATTACTCCTAGAACCGC-3′. These sequences were synthesized as 58 bp stem-loop structures and constructed into pLK0.1-puro vector at *Age*I and *Xba*I sides respectively. Lentiviruses were generated by cotransfection of subconfluent 293T cells, with one of the above recombinant plasmids and packaging plasmids (p△8.2 and pVSVG) by calcium phosphate transfection. To collect infectious lentiviruses, supernatants were centrifuged to remove cell debris and filtered through 0.45 μm filters (Merck Millipore). A549 cells were transduced with the Lentiviruses expressing wtGnT-V, △cGnT-V, shRNA against human GnT-V or scramble non-target shRNA. H1975 cells were transduced with lentiviruses containing GnT-V. For selecting the stable cells, puromycin (2 μg/ml) was added in cells after virus infections. For control transfection, the same protocol was performed with only the empty lentivirus expression vector.

### Immunofluorescence staining and confocal microscopy

Cells were grown on glass coverslips in a 24-well plate, washed with cold PBS, fixed with 4% paraformaldehyde for 30 min. on ice and permeabilized with 0.1% Triton X-100 in PBS for 10 min. on ice. After blocking with 3% BSA in PBS, Coverslips were incubated with respective primary antibodies at 1:100 dilutions with 3% BSA in PBS overnight at 4°C. After washing three times with 3% BSA in PBS, Coverslips were incubated with fluorescein-conjugated secondary antibodies or F-actin-specific dye phalloidin at 1:500 dilutions with 3% BSA in PBS for 1 hr. Cells were then washed three times with PBS, mounted with medium containing 4,6-diamidino-2-phenylindole (DAPI; Vector Laboratories), analysed by using fluorescence microscopy (Olympus, Tokyo, Japan) or laser confocal microscope (Leica LAS AF Lite, Wetzlar, Germany).

### Cell migration and invasion assays

Cells pre-treated with or without TGF-β1 for 24 hrs (GnT-V knockdown) or 48 hrs (GnT-V overexpression), harvested with 0.25% Trypsin, washed twice with RPMI 1640 medium, and resuspended with serum-free medium containing TGF-β1 or BSA at a density of 5 × 10^5^ (A549) or 1 × 10^5^ (H1975) cells/ml. For transwell migration assay, transwell was coated only on the bottom side with 10 μg/ml fibronectin (FN; Sigma-Aldrich, St. Louis, MO, USA) at room temperature for 1 hr; 200 μl of the cell suspension was plated in the upper chamber of a non-coated transwell insert. In the lower chamber, 500 μl of medium with 5% FBS was used as a chemoattractant to encourage cell migration.

For the transwell matrigel invasion assay, the bottom side of the transwell was coated with 10 μg/ml FN at room temperature for 1 hr, the upper chamber of the transwell insert was coated with 80 μl of 2.0 μg/ml matrigel, and 200 μl of the cell suspension was plated in the upper chamber of the matrigel-coated transwell insert. The lower chamber was filled with 500 μl of medium with 5% FBS.

Cells of both assays were incubated at 37°C in 5% CO_2_ for 12 hrs. After incubation, those cells that did not migrate or invade were removed by scraping with a cotton swab. The membrane in the transwell was fixed with 4% paraformaldehyde for 30 min., dyed with 2.5% crystal violet staining for 15 min. and the cells that migrated to lower side under a light microscope were counted. We selected five random fields per filter to count the cells and each independent experiment was repeated at least three times.

### Transfection and dual-luciferase assay

Cell transfection and dual-luciferase assay were performed as previously described 30. Cells cultured in 48-well plates were transfected with Smad-driven-transcriptional luciferase and renilla reporter constructs. The following day, cells were serum-starved and exposed to TGF-β1 for 24 hrs; thereafter, cells were lysed and luciferase activity was measured by using the dual-luciferase reporter assay system of Promega (Madison, WI, USA).

### Statistical analysis

Quantitative data from at least three experiments were expressed as means ± SD. Statistical significance was determined by Student's *t*-test. Differences were considered statistically significant at *P* < 0.05. The *P*-value of the groups in comparison were marked in the Figures by using asterisks, and indicated in the Figure legends as *: *P* < 0.05, **: *P* < 0.01, ***: *P* < 0.001.

## Results

### GnT-V expression is correlated with epithelial identities positively and mesenchymal identities negatively in human lung cancer

**Figure 1:**
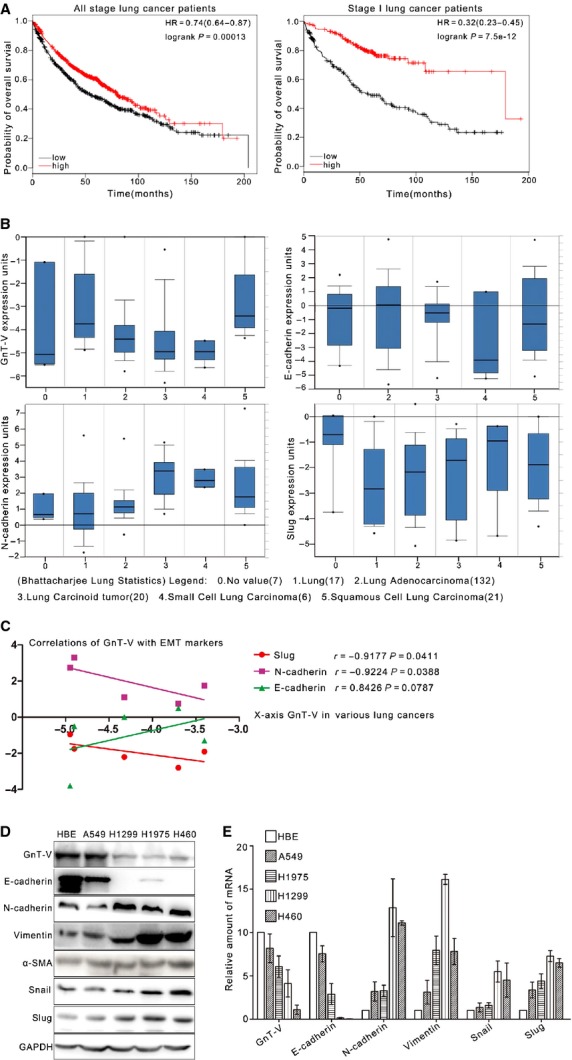
High GnT-V expression is correlated with good prognosis and negatively correlated with EMT in human lung cancer. (**A**) Correlation between higher GnT-V (Affymetrix ID: 212098-at) expression and good overall survival (OS) in all stage (*n* = 1406; left) or stage I (*n* = 440; right) lung cancer patients. Plots were generated online by using a Kaplan–Meier Plotter based on signal intensity in microarray gene expression data from patients for whom OS data are available (www.kmplot.com/lung). Upper curve, red, indicates higher than median expression, and lower curve, black, lower than median expression. (**B**) Oncomine box plot RNA expression data for GnT-V and EMT markers (E-cadherin, *N*-cadherin and Slug) in normal tissue compared with cancer were shown within the Bhattacharjee lung database of publicly available Oncomine microarray data sets (www.oncomine.org). The log2 median-centred ratios for gene expression level are depicted in box-and-whisker plots. Dots represent maximum and minimum outliers from the main data set. For each plot, the following pathological subtypes were evaluated separately. 0, No value (*n* = 7); 1, Lung (*n* = 17); 2, Lung adenocarcinoma (*n* = 132); 3, Lung carcinoid tumour (*n* = 20); 4, Small cell lung carcinoma (*n* = 6); 5, Squamous cell lung carcinoma (*n* = 21). (**C**) Correlations between GnT-V and EMT markers' (E-cadherin, *N*-cadherin and Slug) expression in human lung cancer were analysed by using Bhattacharjee lung database of publicly available Oncomine microarray data sets (www.oncomine.org). (**D** and **E**) Protein (**D**) and mRNA (**E**) expression of GnT-V and EMT markers (E-cadherin, *N*-cadherin, Vimentin, α-SMA, Snail and Slug) in human normal lung cell line HBE and lung cancer cell lines, including A549, H1299, H1945 and H460, was determined by western blot and qRT-PCR respectively.

To study the relationship between GnT-V expression and clinicopathological features of the patients with lung cancer, a Kaplan–Meier survival plot was generated and significance was computed. The tool can be accessed online at www.kmplot.com 25. We used this integrative data analysis tool to validate the prognostic power of GnT-V (Affymetrix ID: 212098-at) in lung cancer by using overall survival. We found that the lung cancer patients with high GnT-V expression had significantly longer survival time than those with low GnT-V expression in all stage lung cancer patients (*n* = 1406; Fig.1A left). In addition, we had also run the analysis for predicting overall survival in stage I lung cancer patients (*n* = 440) alone (Fig.1A right), and the result is similar. These findings are consistent with what Hirotoshi Dosaka-Akita reported 13, suggesting that GnT-V might be considered as a prognostic factor of the patients with lung cancer. Furthermore, to investigate the relevance of GnT-V expression to EMT, the mRNA levels of GnT-V and EMT markers were analysed in Bhattacharjee lung database including 186 lung tumour specimens and 17 normal lung specimens from Oncomine gene expression microarray data sets (www.oncomine.org) 26. The levels of GnT-V mRNA were lower in lung adenocarcinomas, lung carcinoids, small cell lung carcinomas, except squamous cell lung carcinomas, than those in normal counterparts, whereas mRNA levels of *N*-cadherin and Slug were relatively higher in lung cancers as compared with normal counterparts (Fig.1B). The relationship between GnT-V and EMT markers was analysed in mRNA levels. GnT-V mRNA expression was negatively correlated with mesenchymal marker *N*-cadherin and Slug, a repressor inhibiting the E-cadherin expression, and tended to be positively related to epithelial marker E-cadherin mRNA expression (Fig.1C).

Furthermore, one normal lung cell line and four human lung cancer cell lines were subjected to the analysis of mRNA and protein levels of GnT-V and EMT markers by qRT-PCR and western blot. As shown in Figure1D, in HBE, a lung normal bronchial epithelial cell line, and A549, a lung adenocarcinoma cell line, GnT-V protein levels were relatively high, which were correlated with E-cadherin expression positively and *N*-cadherin/Vimentin expression negatively. Meanwhile, we also observed those in lung adenocarcinoma H1975 and H1299, and large cell lung cancer H460 cell lines. The results showed that low expression of GnT-V was closely associated with relative lower E-cadherin and higher *N*-cadherin/Vimentin protein levels. Consistently, the protein levels of Snail and Slug in H1975 and H460 were significantly higher than those of A549 and HBE (Fig.1D). In addition, similar results also showed the close correlation between GnT-V and EMT markers in mRNA levels, which were determined in lung cancer cell lines by qRT-PCR (Fig.1E). These results implicate that in lung cancer cells, GnT-V expression may negatively correlate with EMT behaviour.

### Alterations of β1,6-GlcNAc branched *N*-glycans and GnT-V during TGF-β1-induced EMT in human lung cancer

**Figure 2:**
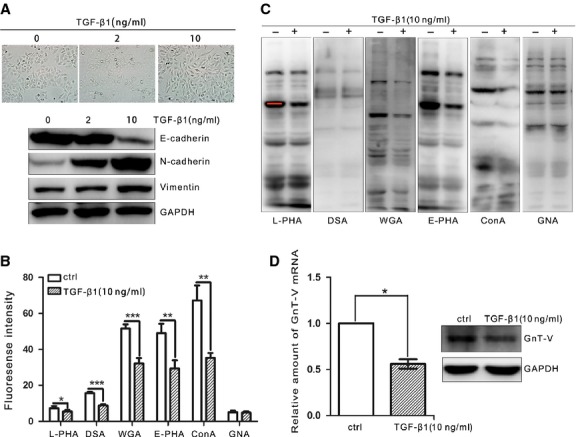
Expression of β1,6-GlcNAc branched *N*-glycans and GnT-V is down-regulated after TGF-β1 treatment in human lung adenocarcinoma A549 cells. A549 cells were cultured to confluency in six-well plates, and treated with TGF-β1 (2 or 10 ng/ml) for indicated 48 hrs. (**A**) Cell morphological changes were observed under microscopy (top) and the protein levels of EMT markers including E-cadherin, *N*-cadherin and Vimentin were examined by western blot (bottom). (**B**) The cell surface *N*-glycans were quantified by flow cytometry with biotinylated L-PHA, DSA, WGA, E-PHA, ConA and GNA lectins, followed by incubation with FITC-conjugated streptavidin. The binding specificity of the lectins used was as indicated: L-PHA, complex-type *N*-glycans containing β1,6-GlcNAc branched structure; DSA, tri-and tetra-antennary complex type of *N*-glycans; WGA, tri-and tetra-antennary complex type of *N*-glycans; E-PHA, bisected type of *N*-glycans; ConA, hybrid and bi-antennary type of *N*-glycans; GNA, high-mannose type of *N*-glycans. (**C**) The *N*-glycans in cell total lysates were detected by L-PHA, DSA, WGA, E-PHA, ConA and GNA lectin blot. GAPDH was used as a load control. (**D**) TGF-β1 reduces GnT-V expression in A549 cells. mRNA (left) and protein (right) expression of GnT-V was determined by qRT-PCR and western blot respectively.

To explore the GnT-V expression and its biological functions during EMT, TGF-β1-induced EMT, a widely admitted EMT model, was employed. After TGF-β1 treatment, the islands of A549 cells turned from the cobblestone-like epithelial morphology into a diffused spindle-like mesenchymal phenotype in a dose-dependent manner, which become very pronounced after 10 ng/ml TGF-β1 treatment (Fig.2A top). TGF-β1 treatment also significantly reduced the E-cadherin protein levels, but increased the *N*-cadherin at the same time (Fig.2A bottom).

Next we sought to identify the effect of TGF-β1 treatment on changes in *N*-glycans' structure and branching status in A549 cells by using lectin-FASC methods. The results showed that hybrid and bi-antennary complex types of *N*-glycans, which could be specially bound by ConA lectin 31, were significantly attenuated during TGF-β1-induced EMT process in A549 cells. In contrast, the high-mannose type of *N*-glycans, which could be detected by GNA binding 32, seemed to be unchanged. L-PHA, DSA and WGA lectins could strongly recognize tri-and tetra-antennary complex type of *N*-glycans, especially L-PHA preferentially recognizes β1,6-GlcNAc branched *N*-glycans by which the cell surface β1,6 GlcNAc branching could be measured 33. Our results showed that *N*-glycans' branching, especially GnT-V's products of β1,6-GlcNAc branched *N*-glycans, was dramatically suppressed by TGF-β1 treatment (Fig.2B). The similar changes of each type of *N*-glycan by lectin blot were detected in A549 cells during EMT (Fig.2C).

To explore whether the reduction of β1,6-GlcNAc branched *N*-glycans was because of the down-regulation of glycosyltransferase, the expression of GnT-V was further studied. It was found that the GnT-V mRNA levels were decreased by about 45% in TGF-β1-treated A549 cells as compared with control cells by qRT-PCR (Fig.2D left). Meanwhile, a significant decrease of GnT-V protein levels was also observed by western blot (Fig.2D right). These results demonstrate that the expression of GnT-V, as well as the formation of β1,6-GlcNAc branched *N*-glycans, is fundamentally suppressed in TGF-β1-induced EMT process.

### Inhibition of β1,6-GlcNAc branched *N*-glycans' formation in lung cancer cells enhances TGF-β1-induced EMT, cell migration and invasion

**Figure 3:**
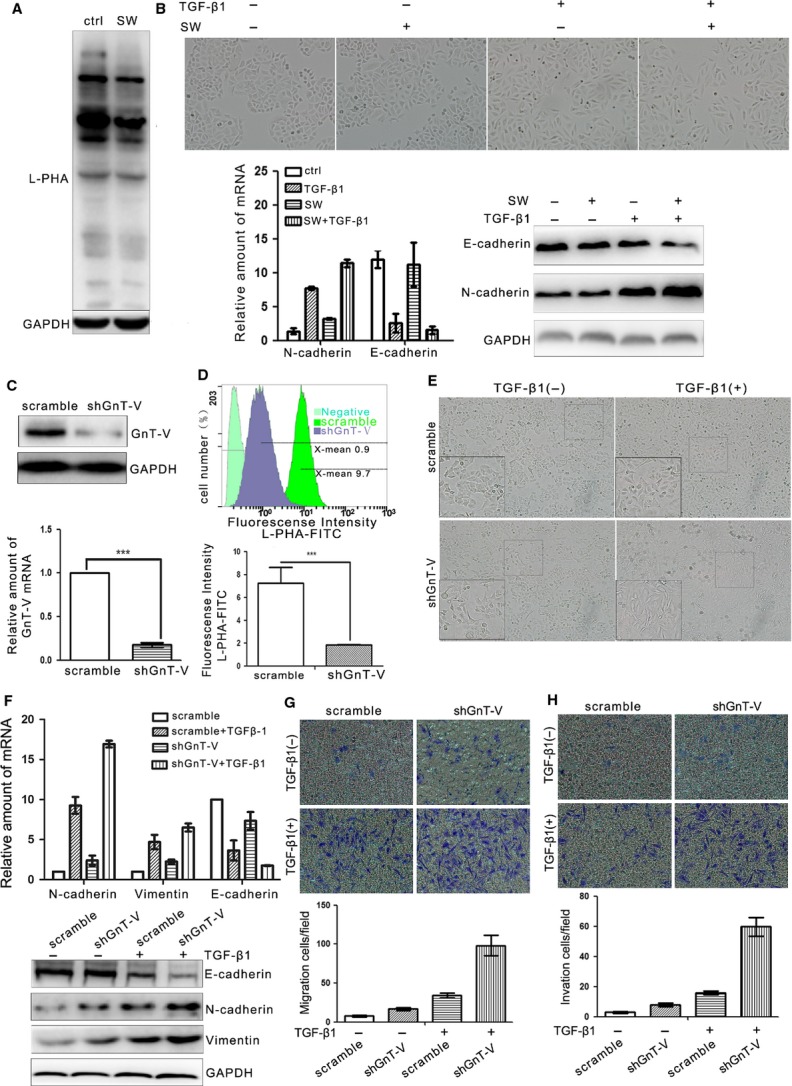
Inhibition of β1,6-GlcNAc branched *N*-glycans' formation through swainsonine treatment or GnT-V knockdown in A549 cells enhances TGF-β1-induced EMT, cell migration and invasion. (**A**) β1,6-GlcNAc branched *N*-glycans were detected by L-PHA lectin blot in A549 cells with or without swainsonine (SW; 1 μg/ml) treatment for 24 hrs, GAPDH was detected as loading control. (**B**) Swainsonine treatment enhances TGF-β1 (5 ng/ml; 24 hrs)-induced EMT in A549 cells, as determined by cell morphological changes (top) and the expression levels of EMT markers (E-cadherin, *N*-cadherin) by qRT-PCR (left bottom) and western blot (right bottom). A549 cells were treated with TGF-β1 (5 ng/ml) alone or with swainsonine (1 μg/ml) for 24 hrs. Cells were pre-treated with swainsonine (1 μg/ml) for 24 hrs before exposure to TGF-β1. (**C**) GnT-V knockdown stable A549 cells were established by using lentiviral shRNA specific for GnT-V (GnT-V shRNA) system and selected by using puromycin. The GnT-V mRNA (bottom) and protein (top) levels in shGnTV and scramble cells were determined by qRT-PCR and western blot respectively. (**D**) The cell surface β1,6-GlcNAc branched *N*-glycans of GnT-V knockdown stable A549 cells were quantified by flow cytometry with L-PHA lectin, the negative control showing the background fluorescence. A representative result of three independent experiments (top) and statistical data (bottom) was presented (bottom). (**E** and **F**) Knockdown of GnT-V in A549 cells enhances TGF-β1 (5 ng/ml; 24 hrs)-induced EMT, as determined by the cell morphological changes (**E**) and the mRNA (**F**, top) and protein (**F**, bottom) levels of EMT markers detected by qRT-PCR and western blot. (**G**) Knockdown of GnT-V in A549 cells increases TGF-β1 (5 ng/ml; 24 hrs)-induced cell migration, as determined by transwell assay (see Materials and methods). A representative result of three independent experiments (top) and statistic data (bottom) were presented. (**H**) Knockdown of GnT-V in A549 cells increases TGF-β1 (5 ng/ml; 24 hrs)-induced cell invasion, as determined by transwell assay with matrigel (see Materials and methods). A representative result of three independent experiments (top) and statistic data (bottom) were presented.

The relationship of GnT-V with EMT markers and the alteration of GnT-V during TGF-β1-induced EMT suggest that GnT-V and its products β1,6-GlcNAc branched *N*-glycans play an important role in the control of EMT in human lung cancer. As there is no previous report showing the role of GnT-V in EMT in human lung cancer, it is important to elucidate whether GnT-V functions in EMT process. First, the effect of the *N*-glycosylation produced by GnT-V on TGF-β1-induced alterations of EMT was explored in A549 cells. After A549 cells were treated with swainsonine, which inhibits Golgi α-mannosidase II and ultimately causes the inhibition of β1,6-GlcNAc branched *N*-glycans' formation, the lectin blot was performed. The result revealed that the β1,6-GlcNAc branched *N*-glycans by L-PHA lectin blot showed a significant decrease (Fig.3A), indicating a dramatic suppression of β1,6-GlcNAc branched *N*-glycans in A549 cells, as expected. And it was surprised that the β1,6-GlcNAc branched *N*-glycans down-regulated by swainsonine significantly enhanced the change of cell morphology in TGF-β1-induced EMT and altered the levels of E-cadherin and *N*-cadherin (Fig.3B). It suggests that β1,6-GlcNAc branched *N*-glycans function as an EMT suppressor.

Given interference synthesis of β1,6-GlcNAc branched *N*-glycans by swainsonine could enhance TGF-β1-induced EMT, we further tried to explore whether direct inhibition of the β1,6-GlcNAc branched *N*-glycans' formation achieved through suppression of GnT-V expression could also enhance TGF-β1-induced EMT. Stable GnT-V knockdown A549 cells were gained in a puromycin selection by using the lentiviral shRNAs system. As shown in Figure3C, in shGnT-V A549 cells, GnT-V mRNA and protein levels were significantly decreased as compared with scramble A549 cells. Consistent with decreased GnT-V mRNA and protein levels, GnT-V knockdown cells showed a dramatically reduced binding with L-PHA by flow cytometry (Fig.3D). Taken together, these results demonstrate that both GnT-V and its products β1,6-GlcNAc branched *N*-glycans are significantly suppressed by shGnT-V expression, and reduction of β1,6-GlcNAc branched *N*-glycans is because of the down-regulation of glycosyltransferase.

Furthermore, we observed the changes of cell morphology and mobility after GnT-V knockdown. GnT-V down-regulation by shRNA induced a scattered distribution of cells and significantly enhanced TGF-β1-induced morphological alterations of EMT, supporting the conclusion that GnT-V functions as an EMT suppressor (Fig.3E) 34. The change of phenotype was associated with clear alterations of TGF-β1-induced EMT markers, E-cadherin was lost to a much greater extent and *N*-cadherin was increased much more in GnT-V knockdown cells as compared with scramble cells (Fig.3F). These results suggest that GnT-V is critical in maintaining the epithelial phenotype and suppressing the EMT of A549 cells. Moreover, TGF-β1-induced EMT is known to be closely related to enhancement of cell motility. To examine whether GnT-V expression affected TGF-β1-induced cell motility, transwell and transwell matrigel assays were employed to detect the cell migration and invasion. First, in the transwell assay, it was observed that more cells migrated to the lower surface of the membrane in the GnT-V knockdown cells after TGF-β1 treatment (Fig.3G). Consistent with this result, in transwell matrigel assay, the invasive ability of GnT-V knockdown cells was also greater enhanced as compared with scramble cells after treatment with TGF-β1 (Fig.3H).

Taken together, these results indicated that TGF-β1-induced EMT, cell migration and invasion in the lung cancer cells were enhanced by interference of β1,6-GlcNAc branched *N*-glycans' formation or suppression of GnT-V expression. It suggests that GnT-V and its products β1,6-GlcNAc branched *N*-glycans are involved in an EMT-like switch facilitating cell migration and invasion *in vitro*.

### Overexpression of GnT-V in lung cancer cells reduces TGF-β1-induced EMT, cell migration and invasion

**Figure 4:**
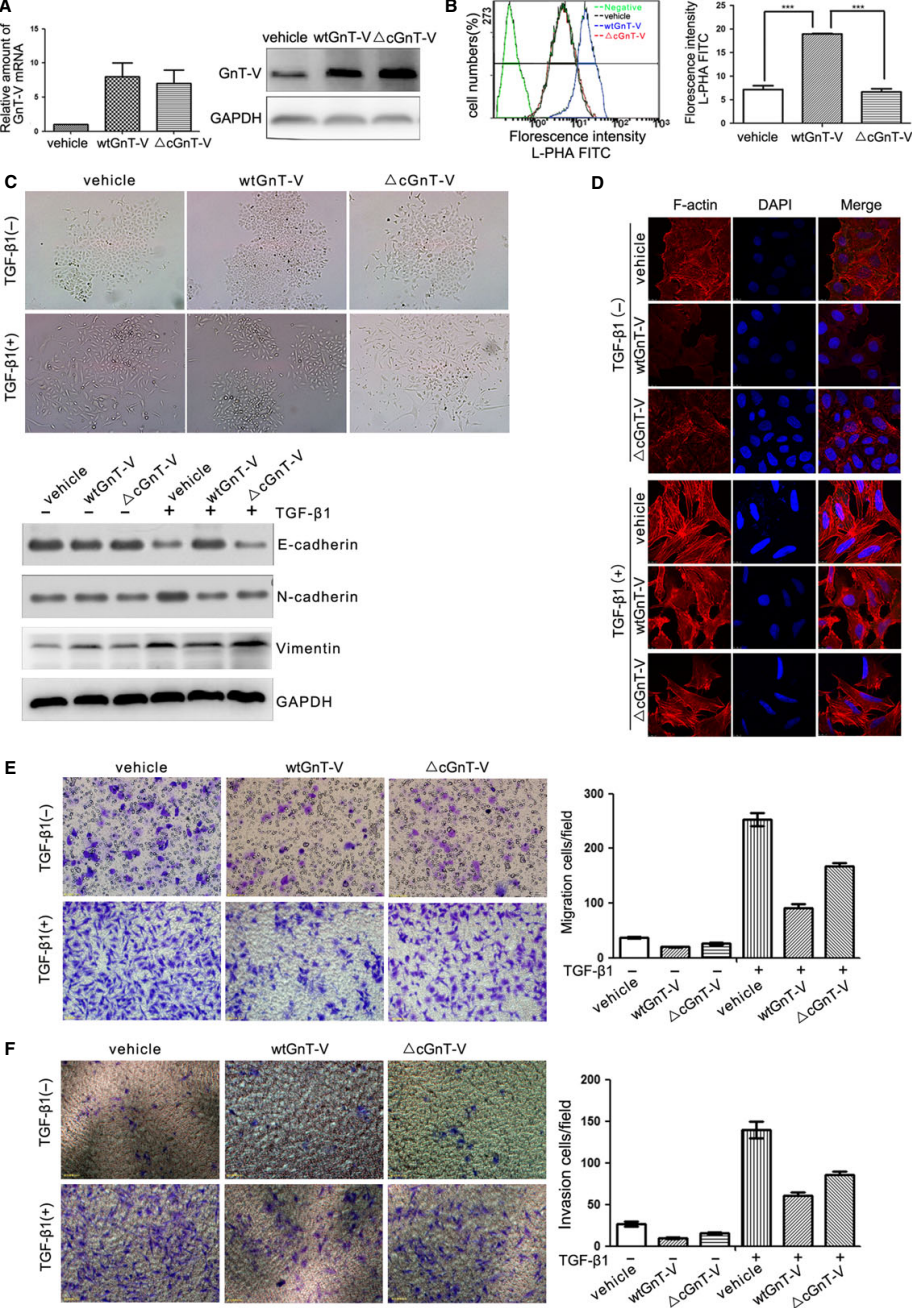
Overexpression of GnT-V in A549 cells suppresses TGF-β1-induced EMT, cell migration and invasion in a catalytic activity–dependent manner. (**A**) GnT-V overexpression stable A549 cells were established by using lentiviral gene specific for wild-type GnT-V (wtGnT-V) or inactive mutant GnT-V (△cGnT-V) system and selected by using puromycin. The GnT-V mRNA (left) and protein (right) levels in stable cells were determined by qRT-PCR and western blot respectively. (**B**) The cell surface β1,6-GlcNAc branched *N*-glycans of GnT-V overexpression stable A549 cells were quantified by flow cytometry with L-PHA lectin, the negative control showing the background fluorescence. A representative result of three independent experiments (left) and statistic data (right) were presented. (**C** and **D**) Overexpression of GnT-V in A549 cells suppresses TGF-β1 (5 ng/ml; 48 hrs)-induced EMT in a catalytic activity–dependent manner, as determined by the cell morphological changes (**C**, top), the expression levels of EMT markers (E-cadherin and *N*-cadherin) by western blot (**C**, bottom) and the actin remodelling by immunofluorescence staining the F-actin (**D**). (**E**) Overexpression of GnT-V in A549 cells suppresses TGF-β1 (5 ng/ml; 48 hrs)-induced cell migration in a catalytic activity–dependent manner, as determined by transwell assay. A representative result of three independent (left) and statistic data (right) were presented. (**F**) Overexpression of GnT-V in A549 cells suppresses TGF-β1 (5 ng/ml; 48 hrs)-induced cell invasion in a catalytic activity–dependent manner, as determined by transwell assay with matrigel. A representative result of three independent experiments (left) and statistic data (right) were presented.

As overexpression of GnT-V is an effective tool to alter the cell surface β1,6-GlcNAc branched *N*-glycans, next we used GnT-V overexpressed A549 cells to investigate in detail the relationship of GnT-V's catalytic activity with TGF-β1-induced EMT. To explore whether GnT-V affecting A549 cells' EMT behaviour was dependent on its catalytic activity, either wild-type GnT-V (wtGnT-V) or inactive mutant GnT-V (△cGnT-V) was transfected stably into A549 cells. The inactive mutant GnT-V, constructed the shortened GnT-V gene without coding sequence for six amino acids at C-terminal extreme end for destroying its catalytic activity. Because the deletion of 4–8 amino acids at its carboxyl-terminus can destroy its catalytic activity 35,36. The stable cells were selected with puromycin and identified as shown in Figure4A. The GnT-V mRNA and protein levels were significantly increased in either wtGnT-V-A549 or △cGnT-V-A549 cells, and a significant enhancement of L-PHA binding in wtGnT-V-A549 cells, whereas little change in L-PHA binding was observed in △cGnT-V-A549 cells as compared with vehicle cells, indicating that △cGnT-V-A549 cells mainly expressed the inactive form of GnT-V protein (Fig.4B). Therefore, these results suggest that β1,6-GlcNAc branched *N*-glycans are prevalent under the increased catalytic activity of GnT-V, and glycosyltransferase activity is deficient in △cGnT-VA549-cells as compared with wtGnT-V-A549 and vehicle cells.

Overexpression of wtGnT-V abolished the gain of a strikingly a mesenchymal phenotype and significantly reduced TGF-β1-induced EMT in A549 cells, as shown by the inhibition of the cell morphological changes (Fig.4C top), the increase of *N*-cadherin and the decrease of E-cadherin (Fig.4C bottom), and the actin remodelling (Fig.4D), supporting the conclusion that GnT-V functions as an EMT suppressor. Consistent with the above results, wtGnT-V overexpression also weakens the ability of TGF-β1-induced cell migration and invasion in A549 cells (Fig.4E and F). Conversely, there was little effect of △cGnT-V overexpression on TGF-β1-induced EMT, cell migration and invasion as compared with vehicle cells (Fig.4C, E and F), indicating that the loss of GnT-V catalytic activities did not have the ability to inhibit the TGF-β1-induced EMT and cell motility in A549 cells. These results suggest that the GnT-V suppressing lung cancer EMT behaviour is glycosyltransferase activity–dependent.

**Figure 5:**
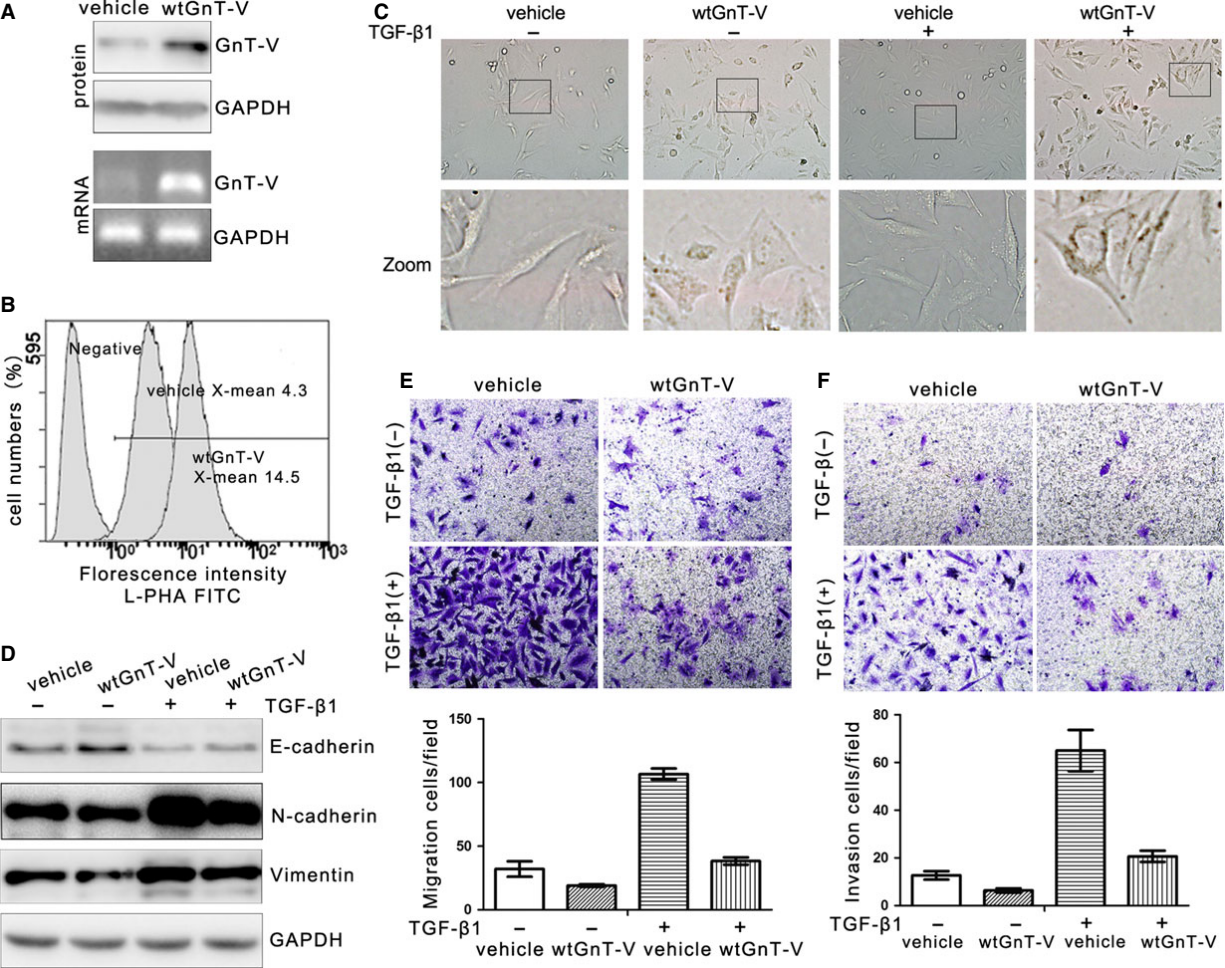
Overexpression of GnT-V reverses EMT and suppresses cell migration and invasion in human lung adenocarcinoma H1975 cells. (**A**) GnT-V overexpression stable H1975 cells were established by using lentiviral gene specific for wild-type GnT-V (wtGnT-V) system and selected by using puromycin. The GnT-V mRNA (bottom) and protein (top) levels in stable cells were determined by RT-PCR and western blot respectively. (**B**) The cell surface β1,6-GlcNAc branched *N*-glycans of GnT-V overexpression stable H1975 cells were detected by flow cytometry with L-PHA lectin, the negative control showing the background fluorescence. (**C**) The cell morphological changes of vehicle and wtGnT-V H1975 cells in response to TGF-β1 (5 ng/ml; 48 hrs) treatment. Photographs were taken of living cells by using a × 20 objective. Insets, representative cell morphology. (**D**) Overexpression of GnT-V in H1975 cells reduces TGF-β1 (5 ng/ml; 48 hrs)-induced EMT, as determined the protein expression levels of EMT markers (E-cadherin and *N*-cadherin) by western blot. (**E**) Overexpression of GnT-V in H1975 cells reduces TGF-β1 (5 ng/ml; 48 hrs)-induced cell migration, as determined by transwell assay. A representative result of three independent experiments (top) and statistic data (bottom) were presented. (**F**) Overexpression of GnT-V in H1975 cells reduces TGF-β1 (5 ng/ml; 48 hrs)-induced cell invasion, as determined by transwell assay with matrigel. A representative result of three independent experiments (top) and statistic data (bottom) were presented.

We also verified whether GnT-V functions as EMT suppressor in H1975, another human lung adenocarcinoma cell line. H1975 cells with stable wtGnT-V overexpression, which originally shows low level of GnT-V expression, exhibit an EMT phenotype, *i.e*. fibroblastoid morphology, high *N*-cadherin and vimentin, and low E-cadherin. As shown in Figure5A and B, wtGnT-V mRNA and protein levels, and L-PHA binding in wtGnT-V-H1975 cells were significantly enhanced as compared with vehicle cells. Strikingly, overexpression of wtGnT-V in H1975 cells partly reversed their mesenchymal phenotype into an epithelial-like one and also the capacity of cell migration and invasion (Fig.5C, E and F). And after TGF-β1 treatment, overexpression of wtGnT-V in these cells also inhibited the TGF-β1-induced EMT, as demonstrated by inhibition of the cell morphological changes, up-regulation of E-cadherin and down-regulation of *N*-cadherin (Fig.5C and D), and suppression of the ability of TGF-β1-induced cell migration and invasion (Fig.5E and F).

Over all, these results suggest that GnT-V plays a role in maintenance of the epithelial phenotype and suppressing the TGF-β1-induced EMT, and cell migration and invasion *via* its product of β1,6-GlcNAc branched *N*-glycans. The target glycoproteins of GnT-V and the underlying mechanisms need further investigation.

### Inhibition of β1,6-GlcNAc branched *N*-glycans' formation enhances the activation of TGF-β/Smads signalling pathway

**Figure 6:**
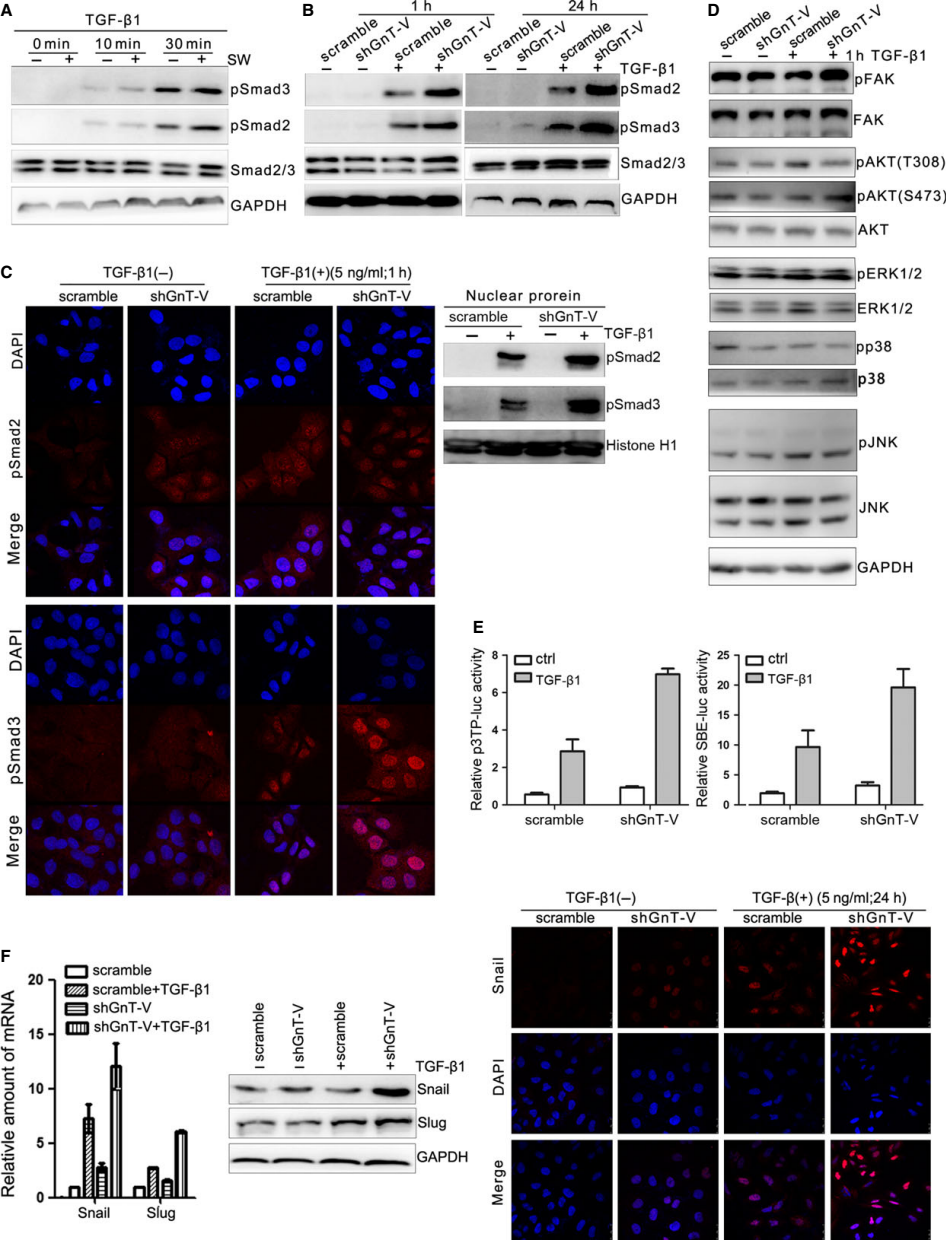
Inhibition of β1,6-GlcNAc branched *N*-glycans' formation through swainsonine treatment or GnT-V knockdown in lung cancer cells enhances the activation of TGF-β/Smads signalling. (**A**) Swainsonine treatment enhances TGF-β1-mediated Smad2 and Smad3 phosphorylation in A549 cells, as determined the Smad2 and Smad3 proteins, and their phosphorylation levels (pSmad2, pSmad3) by western blot. A549 cells were pre-treated with or without swainsonine (1 μg/ml) for 24 hrs, before exposure to TGF-β1 (5 ng/ml) for indicated time periods. (**B**) Knockdown of GnT-V enhances TGF-β1-induced Smad2 and Smad3 phosphorylation in A549 cells. The phosphorylation and total protein levels of Smad2/Smad3 were determined by western blot. Scramble and shGnT-V A549 cells were exposed to TGF-β1 (5 ng/ml) for 1 or 24 hrs. (**C**) Knockdown of GnT-V enhances TGF-β1-induced nuclear translocation of pSmad2 and pSmad3 in A549 cells, as determined by immunofluorescence staining (left) and nuclear protein immunoblotting (right). Scramble and shGnT-V A549 cells were exposed to TGF-β1 (5 ng/ml) for 1 hr. (**D**) Knockdown of GnT-V does not alter TGF-β-non-Smad signalling in A549 cells. Scramble and shGnT-V A549 cells were exposed to TGF-β1 (5 ng/ml) for 1 hr, and the phosphorylation and total protein levels of signalling molecules (FAK, AKT, ERK, p38 and JNK) in TGF-β-non-Smad pathway were determined by western blot. GAPDH was used as loading control. (**E**) Knockdown of GnT-V increases TGF-β1-induced Smad2/Smad3 transcriptional reporter activity in A549 cells, as determined by a dual-luciferase assay. Scramble and shGnT-V A549 cells were transiently transfected by Smad2/4 driven-3TP-luciferase and Smad3/4 driven-(SBE)4-luciferase. After transfection, cells were treated with or without TGF-β1 (5 ng/ml) for another 24 hrs, then dual-luciferase assay was performed. (**F**) Knockdown of GnT-V increases TGF-β1-induced Snail/Slug expression and nuclear translocation in A549 cells, as determined by qRT-PCR (left) and western blot (middle), and immunofluorescence staining (right) of A549 cells' exposure to TGF-β1 (5 ng/ml) for 24 hrs.

It has been known that most of the switches from an epithelial to a mesenchymal-like phenotype are regulated by TGF-β signalling 20. Because both the interference of β1,6-GlcNAc branched *N*-glycans' formation and the knockdown of GnT-V enhance TGF-β1-induced EMT and cell motility in lung cancer A549 cells. Hence, it is speculated that both may regulate some key signal molecules in TGF-β signalling pathway. We found that either swainsonine treatment or GnT-V knockdown of A549 cells caused the increased Smad2 and Smad3 phosphorylation in response to TGF-β1 as compared with control cells (Fig.6A and B). And the results of immunofluorescence staining (Fig.6C left) and nuclear protein immunoblotting (Fig.6C right) showed that shGnT-V-A549 cells' exposure to TGF-β1 had more nuclear translocation of pSmad2 and pSmad3 than scramble cells. In addition to Smad signalling, we also investigated the effect of GnT-V on some TGF-β non-Smad signalling pathways. We detected the phosphorylation of AKT, ERK1/2, P38, JNK and FAK by western blot (Fig.6D). It was found that GnT-V knockdown had little effect on TGF-β-non-Smads signalling except the increased FAK signalling pathway. These results showed that GnT-V knockdown and the inhibition of β1,6-GlcNAc branched *N*-glycans' formation enhanced TGF-β signalling through increased Smads phosphorylation and their nuclear translocation.

Furthermore, we examined the effect of GnT-V on TGF-β1-induced transcriptional activity. As shown in Figure6E, knockdown of GnT-V in A549 cells resulted in significantly enhanced activity of TGF-β1-induced transcriptional response from a Smad2/4-dependent receptor 3TP-luciferase 37 and a Smad3/4-dependent reporter (SBE)_4_-luciferase 38, indicating that GnT-V was involved in the regulation of TGF-β/Smad2/3/4-dependent transcriptional response. It suggests that GnT-V is involved in TGF-β1-induced EMT switch through TGF-β/Smads pathway. Then, to further confirm the effect of GnT-V on Smads-mediated transcriptional activity, we observed the changes of protein and mRNA levels of Snail and Slug, which are strong repressors of E-cadherin expression. Snail and Slug are typical TGF-β downstream target genes, which contain Smad3-binding G/C-rich sequence and are transactivated by Smad3 following TGF-β1 treatment 39. Knockdown of GnT-V enhanced TGF-β1-induced mRNA level of Snail and Slug according to qRT-PCR results (Fig.6F left), which was also confirmed by western blot (Fig.6F middle), and increased nuclear translocation of snail by Immunofluorescence staining (Fig.6F right). All these results demonstrated that knockdown of GnT-V enhanced TGF-β1-induced up-regulation of Smads-mediated transactivation. It suggests that TβRs, one of the GnT-V's substrates, may involve in the process.

### GnT-V impairs the activation of TGF-β/Smads signalling pathway in a catalytic activity–dependent manner

**Figure 7:**
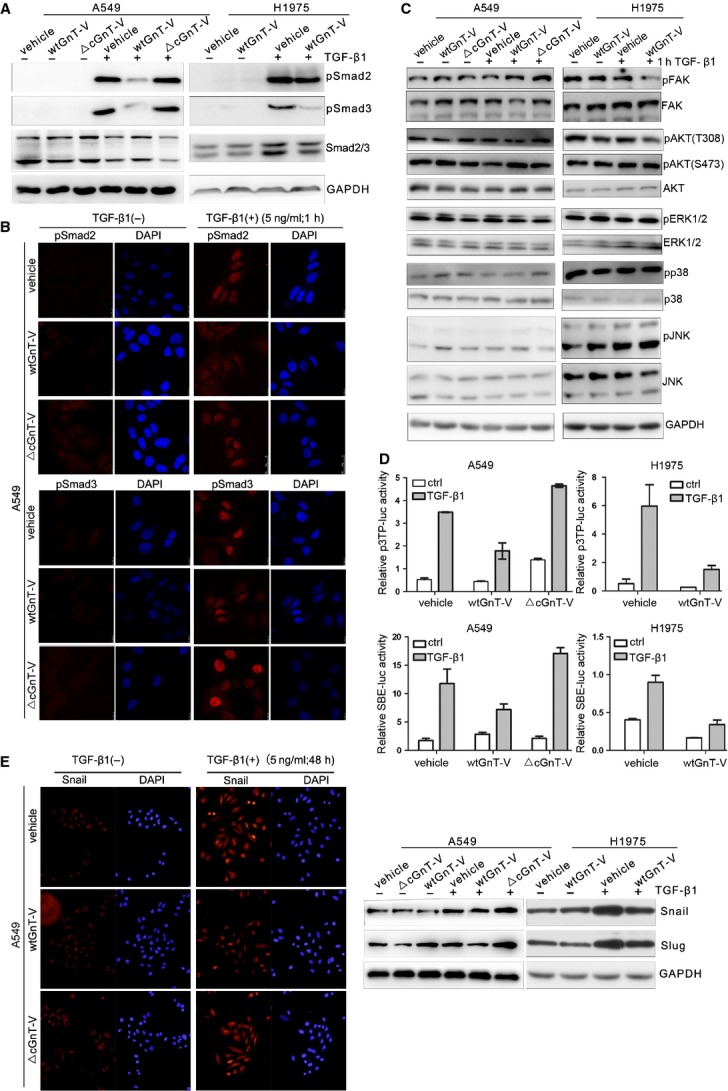
Overexpression of GnT-V in lung cancer cells impairs TGF-β/Smad signalling in a catalytic activity–dependent manner. (**A**) Overexpression of GnT-V in A549 and H1975 cells impairs TGF-β1-induced phosphorylation of Smad2/Smad3 in a catalytic activity–dependent manner. Vehicle A549/H1975 cells, or those stably overexpressing wtGnT-V or △cGnT-V, were treated with TGF-β1 (5 ng/ml) for 1 hr, the phosphorylation and total protein levels of Smad2/Smad3 were measured by western blot by using anti-pSmad2/anti-pSmad3 and anti-Smad2/3 antibody. (**B**) Overexpression of GnT-V in A549 cells prevents TGF-β1-induced nuclear translocation of pSmad2/pSmad3 in a catalytic activity–dependent manner. Vehicle, wtGnT-V and △cGnT-V A549 cells were treated with TGF-β1 (5 ng/ml) for 1 hr, then immunofluorescently stained with anti-pSmad2 /pSmad3 antibody. Nuclei were stained with 4,6-diamidino-2-phenylindole (DAPI). (**C**) Overexpression of GnT-V does not alter TGF-β-non-Smad signalling in A549 and H1975 cells. All cells were treated with TGF-β1 (5 ng/ml) for 1 hr, and signalling molecules including pFAK, FAK, pAKT(Ser473), pAKT(Thr308), pERK, ERK, pP38, P38, pJNK and JNK in TGF-β-non-Smad pathway were determined by western blot. GAPDH was used as loading control. (**D**) Overexpression of GnT-V in A549 and H1975 cells inhibits TGF-β1-induced Smad2/Smad3 transcriptional reporter activity in a catalytic activity–dependent manner, as determined by a dual-luciferase assay. Vehicle A549/H1975 cells, or those stably overexpressing wtGnT-V or △cGnT-V, were transiently transfected by Smad2/4 driven-3TP-luciferase and Smad3/4 driven-(SBE)4-luciferase. After luciferase reporter vectors' transfection, cells were treated with or without TGF-β1 (5 ng/ml) for another 24 hrs, then dual-luciferase assay was performed. (**E**) Overexpression of GnT-V in A549 and H1975 cells reduces TGF-β1-induced Snail/Slug expression and nuclear translocation levels in a catalytic activity–dependent manner, as determined by western blot (right) and immunofluorescence staining (left) of A549 and H1975 cells exposed to TGF-β1 (5 ng/ml) for 48 hrs.

Next, we considered whether the effect of GnT-V overexpression on TGF-β/Smad signalling is not associated with its catalytic activity. Indeed, overexpression of wtGnT-V in A549 cells decreased Smad2 and Smad3 phosphorylation and nuclear translocation of pSmad2/3 in response to TGF-β1 as compared with △cGnT-V and vehicle cells (Fig.7A and B), and the similar results obtained in H1975 cells with stably overexpression of wtGnT-V (Fig.7A). In contrast, overexpression of △cGnT-V, an inactive form, in A549 cells showed no influence on the phosphorylation and nuclear translocation of Smad2 and Smad3 in response to TGF-β1 as compared with vehicle cells (Fig.7A and B). Moreover, in A549 cells, overexpression of wtGnT-V in A549 cells had no significant influence on the phosphorylation levels of AKT, ERK1/2, P38, JNK, except decreased phosphorylation of FAK as compared with △cGnT-V or vehicle cells with TGF-β1 treatment (Fig.7C). These results indicated that GnT-V suppressed TGF-β signalling at the Smads activation stage, consistent with the effect on EMT behaviour, is catalytic activity-dependent.

Furthermore, overexpression of wtGnT-V in A549 cells and H1975 cells reduced the activities of both Smad2/4-driven 3TP and Smad3/4-driven (SBE)_4_ luciferase transcriptional reporter activity (Fig.7D), and blocked the protein up-regulation of Snail and Slug after TGF-β1 treatment as compared with vehicle cells. Moreover, increased nuclear translocation of Snail disappeared in wtGnT-V-A549 cells with TGF-β1 treatment as compared with vehicle cells, explaining the reduction of TGF-β1-induced EMT (Fig.7E). Meanwhile, consistent with phosphorylation of Smads, in △cGnT-V-A549 cells, there was little change in TGF-β1-induced Smad3/Smad4 transcriptional reporter activity, and Snail/Slug expression as compared with vehicle cells (Fig.7D and E). These results indicate that GnT-V suppressing TGF-β1-induced up-regulation of Smads-mediated transactivation is catalytic activity-dependent.

Overall, our data strongly support the importance of GnT-V in the inhibition of a morphological switch from a resting epithelium-like to a migratory phenotype through regulation of the TGF-β/Smads pathway and is also catalytic activity–dependent. And we speculate that GnT-V may act through β1,6-GlcNAc branched *N*-glycans' modification of TβRs to affect the TGF-β1-induced EMT in lung cancer cells.

## Discussion

In the present study, we investigated the effect of GnT-V expression on EMT and its biological functions, such as cell migration and invasion. It was found that GnT-V act as a good prognostic factor which has lower expression in human lung cancer than in normal lung, and GnT-V expression was negative correlated with EMT mesenchymal markers, we also found that GnT-V expression and its products β1,6-GlcNAc modification of cell surface were altered during TGF-β1-induced EMT in lung cancer cells. These results indicate that GnT-V is an important event in control of EMT. Hence, inhibition of β1,6-GlcNAc branched *N*-glycans' formation, shRNA-mediated knockdown of GnT-V and overexpression of wild-type GnT-V or inactivated GnT-V were employed to specifically examine the role of GnT-V in TGF-β1-induced EMT, cell migration and invasion in lung cancer cells. We found that GnT-V suppressed the TGF-β1-induced EMT and cell migration and invasion, and these functions depended on its glycosyltransferase activity. The data further confirmed that GnT-V could be a suppressor of TGF-β1-induced EMT through β1,6-GlcNAc branched *N*-glycans' glycosylation of its target glycoproteins. To the best of our knowledge, this is the first report to demonstrate that GnT-V expression is inversely related to EMT behaviour in lung cancer cells. GnT-V is involved in lung cancer cells' EMT by regulating TGF-β/Smads and its downstream transcription factors through a catalytic activity–dependent manner.

Some reporters have shown that GnT-V and its products, β1,6-GlcNAc branching *N*-glycans, have positive correlation with cancer malignancy and are strongly linked to tumour metastasis 40,41. However, it is not always the case, as demonstrated by the fact that Dosaka-Akita *et al*. reported that lower expression of GnT-V is associated with a shorter survival and a poor prognosis in Stage I NSCLCs 13. The present study also found that in lung cancer, GnT-V expression was lower than that in normal lung, and was favourable prognosis, as well as was inversely related to EMT behaviours. Moreover, GnT-V expression, as well as β1,6-GlcNAc branched *N*-glycans, was down-regulated during TGF-β1-induced EMT. All these results indicate that the role of GnT-V in cancer progression is tissue-specific, and GnT-V may be involved in the lung cancer cells' EMT process. Therefore, there remains a distinct possibility that TβRs, the target glycoprotein of GnT-V, may play a role in TGF-β1-induced EMT, cell migration and invasion in human lung cancer cells.

It is well known that EMT facilitates tumour metastasis. During EMT process, the epithelial cells are converted to the cells with more ability of migration and invasion, through up-regulation of mesenchymal markers, loss of epithelial markers, reduction of cell–cell adhesion and increase of cell–matrix adhesion. Previous studies have suggested the involvement of GnT-V in regulating cell adhesion, migration and invasion in various cancers, simply by affecting the *N*-glycosylation of cell surface protein receptors, including GFRs, integrins and cadherins 41–43. Notably, most of GnT-V triggered β1,6-GlcNAc modification of glycoproteins as EMT markers or EMT regulators contributes to EMT behaviour 44. And much evidence indicates that EMT is a multi-step process and is induced predominantly by the cellular surface glycoproteins, although the underlying mechanisms remain to be clarified. Moreover, the effect of GnT-V on EMT in lung cancer and its biological function have not yet been elucidated. Our results demonstrated that the reduction of β1,6-GlcNAc branched *N*-glycans by either swainsonine or GnT-V knockdown enhanced TGF-β1-induced EMT and in addition increased the capacities of cell migration and invasion in lung cancer A549 cells. Swainsonine treatment and GnT-V knockdown have been reported to significantly increase cell migration and invasion through down-regulation of β1,6-GlcNAc branched *N*-glycans of α5β1 integrin in human choriocarcinoma cells 45. To further directly prove that GnT-V was a suppressor of EMT, which was associated with its catalytic activity, we established overexpression of wild-type GnT-V and inactive △cGnT-V as a negative control for stable cells. And we further confirmed that GnT-V was a suppressor gene to negatively regulate the EMT behaviour, cell migration and invasion in a catalytic activity–dependent manner. These results were consistent with the effect of GnT-V activity in fibrosarcoma–matrix adhesion, monocyte adhesion trans-endothelial migration, and extravillous trophoblast invasion, although were different from that in breast cancer motility 46,47. These diverse results may be because of the differences in the malignancy of the cells. Furthermore, GnT-V expression is regulated in a tissue-specific manner, and the biological functions of GnT-V expression are different in various tissues and cells, depending on the biological function of target substrate glycoproteins, which can vary in different tissues and cells 48. Up-regulation of GnT-V may contribute to altered biological properties of lung cancer cells by increased synthesis of β1,6-GlcNAc branched *N*-glycans of certain target glycoproteins, resulting in inhibition of TGF-β1-induced EMT and cell motility. All these results indicate that the role of GnT-V in cancer progression is tissue-specific. Therefore, there remains a distinct possibility that other target glycoproteins may play a role in EMT, migration and invasion of human lung cancer cells and the target glycoproteins of GnT-V remain to be determined.

To further investigate the molecular mechanisms by which GnT-V affected the EMT behaviours of lung cancer cell, we investigated the effect of GnT-V activity on TGF-β signalling. GnT-V's catalytic activity had little effect on TGF-β non-Smads signalling except the FAK signalling pathway. But it was associated with the decreased level of Smads phosphorylation, nuclear translocation, Smads-driven transcriptional reporter activity, and Snail/Slug expression of Smads downstream transcription factors to down-regulate TGF-β/Smads signalling. Aberrant modification of β1,6-GlcNAc branched *N*-glycans by GnT-V affects the functions of cell surface receptors and the downstream signalling pathways mediated by these receptors. Previous studies have demonstrated that GnT-V^+/+^ mouse cells sensitized to multiple cytokines increased the Galectin-3 cross-linked GnT-V-modified *N*-glycan on EGFR at the cell surface to delay constitutive endocytosis 3,49. Furthermore, this effect of GnT-V is associated with the number of *N*-glycans (n) of glycoprotein. The number of *N*-glycans is a distinct feature of each glycoprotein and cooperates with the avidities for the galectin lattice of glycoproteins to regulate surface glycoprotein residency levels 50. The effect of β1,6-GlcNAc branched *N*-glycans of TβRI and TβRII, which have a few *N*-glycan sites (NXS/T) (*n* = 1 and *n* = 2), on surface retention, is different with high glycosylation sites of EGFR (*n* = 8) 51,52. In GnT-V^+/+^ tumour cells, lattice avidity is strengthened and promotes endocytosis of low-n receptor TβRs, by positive feedback to high-n receptors having high avidities for galectin lattice because of its stability located on cell surface. Moreover, GnT-V^−/−^ tumour cells display reduced galectin-3 binding to complex *N*-glycans on high-n receptors and increased high-n receptors' mobility on cell membrane and, thus, movement into both caveoli and coated-pits, but limited low-n receptor internalization and maintain downstream signalling sensitivity 53,54. Therefore, we speculated that the inhibitory effect of GnT-V on TGF-β signalling pathway possibly increased endocytosis of TβR through the modification of *N*-glycosylation, resulting in reduced cell surface retention, diminished TGF-β signalling, therefore decreased target gene Snails expression, and blocked EMT behaviours. And the exact mechanism by which GnT-V influences the TGF-β signalling requires further investigation.

In brief summary, this is the first evidence that GnT-V suppresses TGF-β1-induced EMT, cell migration and invasion through its catalytic activity in the human lung cancer, which further suggests that GnT-V and its products β1,6-GlcNAc branched *N*-glycans appear to be responsible for modulating EMT and cancer invasion in lung cancer. These findings support the hypothesis that GnT-V and β1,6-GlcNAc branched *N*-glycans may function as a suppressor for modulating EMT and invasion of lung cancer, and provide a hint for development of a new concept to regulate EMT, which may be a potential new therapy target for lung cancer, and that it can be used as a prognostic marker for lung cancer progression.

## References

[b1] Hakomori S (1989). Aberrant glycosylation in tumors and tumor-associated carbohydrate antigens. Adv Cancer Res.

[b2] Taniguchi N, Yoshimura M, Miyoshi E (1998). Gene expression and regulation of *N*-acetylglucosaminyltransferases III and V in cancer tissues. Adv Enzyme Regul.

[b3] Varki A (1993). Biological roles of oligosaccharides: all of the theories are correct. Glycobiology.

[b4] Gu J, Sato Y, Kariya Y (2009). A mutual regulation between cell-cell adhesion and *N*-glycosylation: implication of the bisecting GlcNAc for biological functions. J Proteome Res.

[b5] Granovsky M, Fata J, Pawling J (2000). Suppression of tumor growth and metastasis in Mgat5-deficient mice. Nat Med.

[b6] Partridge EA, Le Roy C, Di Guglielmo GM (2004). Regulation of cytokine receptors by Golgi *N*-glycan processing and endocytosis. Science.

[b7] Fernandes B, Sagman U, Auger M (1991). Beta 1-6 branched oligosaccharides as a marker of tumor progression in human breast and colon neoplasia. Cancer Res.

[b8] Murata K, Miyoshi E, Kameyama M (2000). Expression of *N*-acetylglucosaminyltransferase V in colorectal cancer correlates with metastasis and poor prognosis. Clin Cancer Res.

[b9] Handerson T, Camp R, Harigopal M (2005). Beta1,6-branched oligosaccharides are increased in lymph node metastases and predict poor outcome in breast carcinoma. Clin Cancer Res.

[b10] Ito Y, Miyoshi E, Sakon M (2001). Elevated expression of UDP-*N*-acetylglucosamine: alphamannoside beta1,6*N*-acetylglucosaminyltransferase is an early event in hepatocarcinogenesis. Int J Cancer.

[b11] Ishimura H, Takahashi T, Nakagawa H (2006). *N*-acetylglucosaminyltransferase V and beta1-6 branching *N*-linked oligosaccharides are associated with good prognosis of patients with bladder cancer. Clin Cancer Res.

[b12] Wang X, He H, Zhang H (2013). Clinical and prognostic implications of beta1, 6-*N*-acetylglucosaminyltransferase V in patients with gastric cancer. Cancer Sci.

[b13] Dosaka-Akita H, Miyoshi E, Suzuki O (2004). Expression of *N*-acetylglucosaminyltransferase v is associated with prognosis and histology in non-small cell lung cancers. Clin Cancer Res.

[b14] Inamori K, Gu J, Ohira M (2006). High expression of *N*-acetylglucosaminyltransferase V in favorable neuroblastomas: involvement of its effect on apoptosis. FEBS Lett.

[b15] Thiery JP (2002). Epithelial-mesenchymal transitions in tumour progression. Nat Rev Cancer.

[b16] Thiery JP, Acloque H, Huang RY (2009). Epithelial-mesenchymal transitions in development and disease. Cell.

[b17] Heldin CH, Landstrom M, Moustakas A (2009). Mechanism of TGF-beta signaling to growth arrest, apoptosis, and epithelial-mesenchymal transition. Curr Opin Cell Biol.

[b18] Huber MA, Kraut N, Beug H (2005). Molecular requirements for epithelial-mesenchymal transition during tumor progression. Curr Opin Cell Biol.

[b19] Yang Y, Pan X, Lei W (2006). Transforming growth factor-beta1 induces epithelial-to-mesenchymal transition and apoptosis *via* a cell cycle-dependent mechanism. Oncogene.

[b20] Polyak K, Weinberg RA (2009). Transitions between epithelial and mesenchymal states: acquisition of malignant and stem cell traits. Nat Rev Cancer.

[b21] Xu J, Lamouille S, Derynck R (2009). TGF-beta-induced epithelial to mesenchymal transition. Cell Res.

[b22] Margadant C, Sonnenberg A (2010). Integrin-TGF-beta crosstalk in fibrosis, cancer and wound healing. EMBO Rep.

[b23] Xu Q, Isaji T, Lu Y (2012). Roles of *N*-acetylglucosaminyltransferase III in epithelial-to-mesenchymal transition induced by transforming growth factor beta1 (TGF-beta1) in epithelial cell lines. J Biol Chem.

[b24] Yu M, Sun J, Thakur C (2014). Paradoxical roles of mineral dust induced gene on cell proliferation and migration/invasion. PLoS ONE.

[b25] Gyorffy B, Surowiak P, Budczies J (2013). Online survival analysis software to assess the prognostic value of biomarkers using transcriptomic data in non-small-cell lung cancer. PLoS ONE.

[b26] Bhattacharjee A, Richards WG, Staunton J (2001). Classification of human lung carcinomas by mRNA expression profiling reveals distinct adenocarcinoma subclasses. Proc Natl Acad Sci USA.

[b27] Rhodes DR, Yu J, Shanker K (2004). ONCOMINE: a cancer microarray database and integrated data-mining platform. Neoplasia.

[b28] Yao W, Yu X, Fang Z (2012). Profilin1 facilitates staurosporine-triggered apoptosis by stabilizing the integrin beta1-actin complex in breast cancer cells. J Cell Mol Med.

[b29] Li GC, Li N, Zhang YH (2009). Mannose-exposing myeloid leukemia cells detected by the sCAR-PPA fusion protein. Int J Hematol.

[b30] Yin P, Zhao C, Li Z (2012). Sp1 is involved in regulation of cystathionine gamma-lyase gene expression and biological function by PI3K/Akt pathway in human hepatocellular carcinoma cell lines. Cell Signal.

[b31] Sumner JB, Gralen N, Eriksson-Quensel IB (1938). The molecular weights of Urease, Canavalin, Concanavalin A and Concanavalin B. Science.

[b32] Shibuya N, Berry JE, Goldstein IJ (1988). One-step purification of murine IgM and human alpha 2-macroglobulin by affinity chromatography on immobilized snowdrop bulb lectin. Arch Biochem Biophys.

[b33] Cummings RD, Kornfeld S (1982). Characterization of the structural determinants required for the high affinity interaction of asparagine-linked oligosaccharides with immobilized Phaseolus vulgaris leukoagglutinating and erythroagglutinating lectins. J Biol Chem.

[b34] Lu Z, Jiang G, Blume-Jensen P (2001). Epidermal growth factor-induced tumor cell invasion and metastasis initiated by dephosphorylation and downregulation of focal adhesion kinase. Mol Cell Biol.

[b35] Korczak B, Le T, Elowe S (2000). Minimal catalytic domain of *N*-acetylglucosaminyltransferase V. Glycobiology.

[b36] Wang L, Liang Y, Li Z (2007). Increase in beta1-6 GlcNAc branching caused by *N*-acetylglucosaminyltransferase V directs integrin beta1 stability in human hepatocellular carcinoma cell line SMMC-7721. J Cell Biochem.

[b37] Wrana JL, Attisano L, Carcamo J (1992). TGF beta signals through a heteromeric protein kinase receptor complex. Cell.

[b38] Zawel L, Dai JL, Buckhaults P (1998). Human Smad3 and Smad4 are sequence-specific transcription activators. Mol Cell.

[b39] Thuault S, Tan EJ, Peinado H (2008). HMGA2 and Smads co-regulate SNAIL1 expression during induction of epithelial-to-mesenchymal transition. J Biol Chem.

[b40] Guo HB, Randolph M, Pierce M (2007). Inhibition of a specific *N*-glycosylation activity results in attenuation of breast carcinoma cell invasiveness-related phenotypes: inhibition of epidermal growth factor-induced dephosphorylation of focal adhesion kinase. J Biol Chem.

[b41] Guo HB, Lee I, Kamar M (2002). Aberrant *N*-glycosylation of beta1 integrin causes reduced alpha5beta1 integrin clustering and stimulates cell migration. Cancer Res.

[b42] Guo HB, Lee I, Kamar M (2003). *N*-acetylglucosaminyltransferase V expression levels regulate cadherin-associated homotypic cell-cell adhesion and intracellular signaling pathways. J Biol Chem.

[b43] Wang C, Yang Y, Yang Z (2009). EGF-mediated migration signaling activated by *N*-acetylglucosaminyltransferase-V *via* receptor protein tyrosine phosphatase kappa. Arch Biochem Biophys.

[b44] Scanlon CS, Van Tubergen EA, Inglehart RC (2013). Biomarkers of epithelial-mesenchymal transition in squamous cell carcinoma. J Dent Res.

[b45] Yamamoto E, Ino K, Miyoshi E (2009). *N*-acetylglucosaminyltransferase V regulates extravillous trophoblast invasion through glycosylation of alpha5beta1 integrin. Endocrinology.

[b46] Guo HB, Lee I, Bryan BT (2005). Deletion of mouse embryo fibroblast *N*-acetylglucosaminyltransferase V stimulates alpha5beta1 integrin expression mediated by the protein kinase C signaling pathway. J Biol Chem.

[b47] Yang HM, Yu C, Yang Z (2012). *N*-acetylglucosaminyltransferase V negatively regulates integrin alpha5beta1-mediated monocyte adhesion and transmigration through vascular endothelium. Int J Oncol.

[b48] Perng GS, Shoreibah M, Margitich I (1994). Expression of *N*-acetylglucosaminyltransferase V mRNA in mammalian tissues and cell lines. Glycobiology.

[b49] Lajoie P, Partridge EA, Guay G (2007). Plasma membrane domain organization regulates EGFR signaling in tumor cells. J Cell Biol.

[b50] Lau KS, Dennis JW (2008). *N*-Glycans in cancer progression. Glycobiology.

[b51] Koli KM, Arteaga CL (1997). Processing of the transforming growth factor beta type I and II receptors. Biosynthesis and ligand-induced regulation. J Biol Chem.

[b52] Zhen Y, Caprioli RM, Staros JV (2003). Characterization of glycosylation sites of the epidermal growth factor receptor. Biochemistry-Us.

[b53] Lau KS, Partridge EA, Grigorian A (2007). Complex *N*-glycan number and degree of branching cooperate to regulate cell proliferation and differentiation. Cell.

[b54] Stanley P (2007). A method to the madness of *N*-glycan complexity?. Cell.

